# Aim-less translation: loss of *Saccharomyces cerevisiae* mitochondrial translation initiation factor mIF3/Aim23 leads to unbalanced protein synthesis

**DOI:** 10.1038/srep18749

**Published:** 2016-01-05

**Authors:** Anton Kuzmenko, Ksenia Derbikova, Roger Salvatori, Stoyan Tankov, Gemma C. Atkinson, Tanel Tenson, Martin Ott, Piotr Kamenski, Vasili Hauryliuk

**Affiliations:** 1 University of Tartu, Institute of Technology, Nooruse 1, 50411 Tartu, Estonia; 2 Molecular Biology Department, Faculty of Biology, M.V. Lomonosov Moscow State University, 1/12 Leninskie Gory, 119991 Moscow, Russia; 3 Department of Biochemistry and Biophysics, Center for Biomembrane Research, Stockholm University, SE-106 91 Stockholm, Sweden; 4 Department of Molecular Biology, Umeå University, Building 6K, 6L University Hospital Area, SE-901 87 Umeå, Sweden; 5 Laboratory for Molecular Infection Medicine Sweden (MIMS), Umeå University, Building 6K and 6L, University Hospital Area, SE-901 87 Umeå, Sweden

## Abstract

The mitochondrial genome almost exclusively encodes a handful of transmembrane constituents of the oxidative phosphorylation (OXPHOS) system. Coordinated expression of these genes ensures the correct stoichiometry of the system’s components. Translation initiation in mitochondria is assisted by two general initiation factors mIF2 and mIF3, orthologues of which in bacteria are indispensible for protein synthesis and viability. mIF3 was thought to be absent in *Saccharomyces cerevisiae* until we recently identified mitochondrial protein Aim23 as the missing orthologue. Here we show that, surprisingly, loss of mIF3/Aim23 in *S. cerevisiae* does not indiscriminately abrogate mitochondrial translation but rather causes an imbalance in protein production: the rate of synthesis of the Atp9 subunit of F_1_F_0_ ATP synthase (complex V) is increased, while expression of Cox1, Cox2 and Cox3 subunits of cytochrome *c* oxidase (complex IV) is repressed. Our results provide one more example of deviation of mitochondrial translation from its bacterial origins.

The mitochondria of eukaryotic cells serve numerous functions; they generate ATP via oxidative phosphorylation; synthesize fatty acids, iron-sulfur clusters and heme, and orchestrate apoptosis, i.e. programmed cell death^1^. According to the endosymbiotic theory, these organelles originate from a free-living bacterium that survived engulfment by an early eukaryote to become an obligate endosymbiont^2^. Most of the mitochondrial genes were subsequently transferred to the nuclear genome, leaving only a handful remaining in the mitochondrial DNA (mtDNA). This very limited set of genes mainly encodes components of the translational apparatus (tRNAs, ribosomal RNAs and, in yeast ribosomal protein Var1) and membrane constituents of the oxidative phosphorylation (OXPHOS) system^3^. These total just 8 proteins in yeast *Saccharomyces cerevisiae*^4^ and 13 in humans^5^.

The very presence of a protein-coding genome, however small, has necessitated the preservation of functional mitochondrial protein synthesis machinery during evolution. The mitochondrial translational apparatus resembles that of its bacterial ancestors^6^. However, over the course of evolution it has undergone significant diversification. Recent high-resolution structures of yeast and mammalian mitochondrial ribosomes have revealed several unusual features: the 5S ribosomal RNA (rRNA) is absent altogether and mt-tRNA^Val^ is found in its place, the 3′ end of the 12S rRNA lacks an anti-Shine-Dalgarno sequence that in bacterial ribosome directs the ribosome to the Shine-Dalgarno element of the mRNA upstream of the start codon, and uniquely to mitchondria a GTPase protein mS29 forms an integral part of the 28S small ribosomal subunit^7^,^8^,^9^,^10^. Nuclear-encoded mitochondrial translational factors assisting the ribosome also differ from the canonical complement: universal bacterial initiation factor IF1 is absent^11^ while a suite of specific accessory factors, so-called translational activators, promote yeast mitochondrial translation in an mRNA-specific manner^12^. Duplication and subsequent divergence of elongation factor EF-Tu in arthropods has led to paralogs that are specialized for delivery of specific tRNA species of highly unusual architecture^13^,^14^, and most eukaryotes carry two copies of EF–G, which have become specialized for one of EF–G’s two roles in ribosome recycling and translocation^14^,^15^. Polypeptide release in human mitochondria is mediated by four release factors–RF1Lmt/mtRF1a, RF1 mt, C12orf65 and ICT1–with the latter being an integral component of the mitochondrial ribosome^16^.

Translation initiation in mitochondria is orchestrated by bacteria-like general initiation factors mIF2 and mIF3, which in yeast are aided by the mitochondria-specific translational activators^17^,^18^,^19^,^20^. For many years, mIF3 was thought to be absent in the yeast *S. cerevisiae*, due to a homologue not being found using standard sequence searching methods. Recently however, identification of *S. cerevisiae* mitochondrial protein Aim23 as an mIF3 orthologue using more sensitive searching and phylogenetic analysis has paved the way for genetic investigations of mIF3 function in this model organism^11^. High-throughput screening assays following yeast growth on non-fermentable media together with determination of petite frequencies have demonstrated that the *aim23* gene is required for mitochondrial functionality, and Aim23 has been hypothesized to be involved in assembly of respiration complexes^21^. Supporting this hypothesis is a lack of observable membrane potential and dramatically decreased oxygen consumption in an *aim23*∆ strain^11^. The mitochondrial defect in the *aim23*∆ strain is complemented by the expression of human^17^ and *Schizosaccharomyces pombe*^11^ mIF3 and–partially–of *Escherichia coli* IF3^17^, demonstrating that Aim23 is a *bona fide* mIF3.

To date the role of mIF3/Aim23 in *S. cerevisiae* mitochondrial translation has not been directly tested. The bacterial homolog of mIF3/Aim23, IF3, is important for tRNA and mRNA selection during translation initiation^22^. In addition to its role in translation initiation, IF3 participates in ribosomal recycling after completion of the polypeptide chain - it prevents re-association of ribosomal subunits dissociated by translational factors EF–G and RRF^23^ and promotes subsequent dissociation of tRNA and mRNA from the small subunit^24^. Given the central role of IF3 in the ribosomal functional cycle it is not surprising that the gene *infC* encoding IF3 in *Escherichia coli* is essential^25^. Moreover, a decrease in IF3 cellular level results in dramatic reduction of the polysomal fraction, indicating abrogation of cellular protein biosynthesis^25^.

In this report we have investigated the role of mIF3/Aim23 in mitochondrial functionality and protein synthesis in yeast *S. cerevisiae*. Surprisingly, mIF3/Aim23 is partially dispensable for mitochondrial functionality and mitochondrial protein synthesis; *S. cerevisiae* lacking the *AIM23* gene can still grow on non-fermentable carbon sources requiring mitochondrial respiration, and the mitochondrial translational system can synthesize the full protein repertoire encoded in the mtDNA. However, the absence of mIF3/Aim23 causes a pronounced misbalance in the relative levels of mitochondrially encoded proteins and significant retardation of growth on non-fermentable media requiring respiration. These results underscore the differences in translation initiation in mitochondria, where only a handful of mRNA molecules are translated with a help of numerous specialized factors, and bacteria, where translation of a vast variety of mRNAs is orchestrated by three essential canonical initiation factors IF1, IF2 and IF3.

## Results

### Effects of AIM23 disruption on *S. cerevisiae* growth on non-fermentable carbon sources and mitochondrial functionality

**Figure 1:**
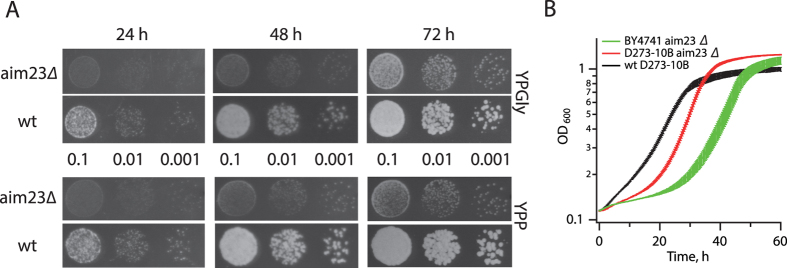
*S. cerevisiae* strains lacking *AIM23* can grown on non-fermentable carbon sources requiring mitochondrial respiration. (**A**) Wild type and *aim23*∆ yeast strains in a D273-10B background^17^ were grown in liquid YPD media until OD_600_ of 3–4, washed with water and spotted at 10× serial dilutions onto solid non-fermentable media: YPGly (glycerol as a carbon source, upper panel) and YPP (ethanol as a carbon source, lower panel). Growth at 30 °C was scored at 24, 48 and 72 hours. (**B**) Wild type D273-10B and *aim23*∆ cells in D273-10B and BY4741 backgrounds were processed as in (**A**) and inoculated into the liquid YPGly media with a starting OD_600_ of 0.1 and growth was monitored at 30 °C for 60 hours. Error bars indicate the standard deviation of the mean of three independent experiments.

We have previously characterized the growth of a *S. cerevisiae aim23*∆ strain on solid media with glycerol as a non-fermentable carbon source–a common test for yeast mitochondrial functionality–and concluded that the strain is incapable of respiration^11^,^17^. However, inspection of plates incubated for 72 hours at 30 °C reveals that the *AIM23*-deficient strain does, eventually, form detectable colonies on both glycerol (Fig. 1A, upper panel) and ethanol (Fig. 1A, lower panel), although growth is significantly retarded in comparison to the parental strain. This growth delay is the likely reason why the phenomenon went unnoticed by us as well as Hess and colleagues who identified *AIM23* as a gene necessary for mitochondrial functionality in earlier high-throughput assays^21^.

The S288C-based BY4741 background we originally used for creating the *aim23*∆ strain^11^ carries several polymorphisms in mitochondrial DNA polymerase *MIP1*, calcium-dependent mitochondrial ADP/ATP carrier *SAL1* and mitochondrial inner membrane protein involved in ubiquinone biosynthesis *CAT5,* which negatively affects the stability of mitochondrial DNA^26^ (mtDNA). To make sure that the observed phenotype of the *aim23*∆ strain is not linked to these polymorphisms, we have recreated *aim23*∆ in a D273-10B background devoid of them^17^. Deletion of *AIM23* leads to growth retardation in liquid YPGly regardless of the strain background (Fig. 1B). The effect is more pronounced in BY4741, possibly due to the cumulative effect of *AIM23* loss on mitochondrial functionality and polymorphisms in *MIP1, SAL1* and *CAT5* destabilizing the mtDNA. The pronounced lag phase of the *aim23*∆ strains could be due to outgrowth on the glycerol media mediated by a fraction of the inoculum carrying secondary compensatory mutations that restore mitochondrial functionality. However, this is unlikely to be the case as the serial dilutions of the *aim23*∆ strain give similar numbers of colonies as the wild type, suggesting that re-growth is not mediated by only a fraction of mutant cells (Fig. 1A). Therefore, we conclude that the lag is caused by slow adaptation of the *aim23*∆ strain to non-fermentable media that requires mitochondrial respiration. Subsequent experiments were performed in a D273-10B background, as its increased mtDNA stability makes it more suitable for investigation of mitochondrial functions.

**Figure 2:**
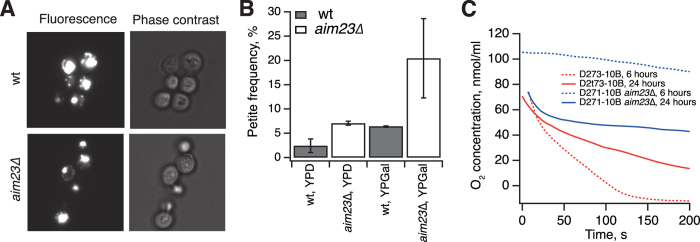
Loss of *AIM23* does not lead to the degradation of mtDNA and abrogation of mitochondrial respiration. The experiments were performed using a congenic set of wild type and *aim23*∆ strains in D273-10B background. Error bars indicate the standard deviation of the mean of at least three independent experiments. (**A**) Nuclear and mitochondrial DNA of yeast cells grown on YPGal media were stained with DAPI according to Amberg *et al.*^28^ and visualized in epi-fluorescence and phase contrast modes. (**B**) Petite frequency as measured by colony count. Yeast cells were grown overnight in either YPD or YPGal liquid media, diluted with fresh media and grown to OD_600_ of 0.8–1.0, washed with water and plated onto solid YPGly media supplemented with 0.1% of glucose. The number of small colonies (“petites”) was scored after 5 days of growth at 30 °C and is expressed as a percentage of the total colony count. (**C**) Oxygen consumption by cells grown on liquid YPGly at 30 °C monitored by a Clark electrode. In wild type cells, O_2_ consumption dropped from 155.4 nmol/ml/min per OD_600_ after 6 hours of growth on glycerol to 48.5 nmol/ml/min per OD_600_ after 24 hours. In *aim23*∆ cells, O_2_ consumption increased from 9.7 nmol/ml/min per OD_600_ after 6 hours of growth on glycerol to 78.5 nmol/ml/min per OD_600_ after 24 hours.

We have characterized the *aim23*∆ D273-10B strain using several functional tests assessing mitochondrial functionality during cell growth on liquid fermentable media supplemented with galactose (YPGal). Galactose, like glucose, is metabolized by fermentation, bypassing the need for mitochondrial respiration; but unlike glucose, it does not suppress mitochondrial function^27^, therefore allowing detection of defects in mitochondrial functionality. Analysis of the pair of congenic wild type and *aim23*∆ strains with phase contrast and fluorescent microcopy using DNA staining with DAPI (4′, 6-diamidino-2-phenylindole)^28^ revealed the presence of mtDNA in both strains (Fig. 2A). This assay cannot, however, distinguish between normal mtDNA and that containing deletions. Such deletions are indicative of underlying defects in mitochondrial function causing mtDNA instability and lead to the formation of small colony variants defective in mitochondrial respiration, so-called ‘petites’^29^,^30^. We assessed the petite frequency in the *aim23*∆ strain using a standard colony count assay^30^ (Fig. 2B). In good agreement with the results of Hess and colleagues^21^, petite incidence in the *aim23*∆ strain is increased approximately three times in comparison to the congenic wild type strain. The effect is present when cells are grown using either glucose or galactose as a carbon source; however, growth on galactose leads to a further increase in the proportion of ‘petites’, indicating unsuppressed defective mitochondrial activity as an underlying cause. To assess mitochondrial functionality directly, we followed oxygen consumption using a Clark-type oxygen electrode^31^. Consumption was measured in yeast cultures pre-grown in liquid culture with a non-fermentable carbon source, glycerol, for 6 and 24 hours (Fig. 2C). These time points correspond to the lag phase and exponential growth of the *aim23*∆ strain, and to exponential phase and consequent cessation of growth of the wild type, respectively (Fig. 1B). In wild type cells the oxygen consumption dropped significantly after 24 hours of growth, coinciding with the end of rapid exponential growth at that point. In the *aim23*∆ strain, O_2_ consumption increased from near-absent after 6 hours of growth on glycerol to levels close to wild type after 24 hours, again in good agreement with the growth measurements.

Taken together, our results demonstrate at least partial mitochondrial functionality in the *aim23*∆ strain. Since mitochondrial protein synthesis is indispensible for mitochondrial functionality, the ability of the *aim23*∆ strain to grow on glycerol and to respire suggests that, surprisingly, yeast mitochondrial translation can operate even in the absence of mIF3/Aim23.

### Effects of AIM23 disruption on *S. cerevisiae* mitochondrial translation and mitochondrial mRNA levels

**Figure 3:**
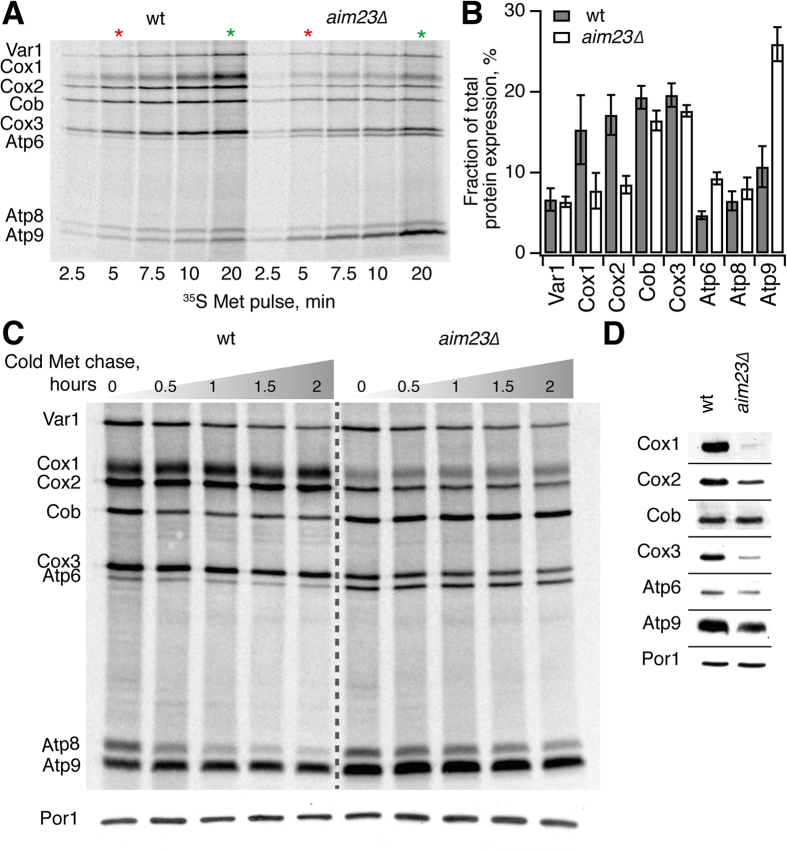
Lack of mIF3/AIM23 leads to unbalanced synthesis of proteins encoded in mtDNA. The experiments were performed using a congenic set of wild type and *aim23*∆ strains in D273-10B background. (**A**) Time course of ^35^S-methionine incorporation in mitochondrially synthesized proteins in live yeast cells. Cytoplasmic translation was suppressed by the addition of 0.2 mg/ml cycloheximide as per Gouget and colleagues^32^. 5 minutes (red asterisk) and 20 minutes (green asterisk) time points were used for quantitative analysis of relative protein expression presented on Fig. 3B and Supplementary Figure 2, respectively. (**B**) Levels of mitochondrially-encoded proteins after 5 min labeling with ^35^S-methionine. The relative expression is normalized to total expression of mitochondrially encoded protein genes. Error bars indicate the standard deviation of the mean of at least three independent experiments.(**C**) Turnover of mitochondrially synthesized proteins in wild type and *aim23*∆ strains. After 15 minutes of ^35^S methionine pulse labeling was carried out as per Gouget and colleagues^32^, the labeling reaction was stopped by the addition of cold methionine (final concentration of 80 mM) and puromycin (final concentration of 4 μg/ml). Samples were collected after the indicated time points, proteins were resolved on SDS PAGE and visualized by radioautography. Western blot detection of Porin 1 (Por1) was used as a control for equal loading. Two additional biological replicates of the experiment are presented as a Supplementary Figure 3. (**D**) Western blot analysis of steady-state levels of mitochondrial proteins in wild-type and *aim23*∆ strains. Cells were grown until OD_600_ ≈ 3.0 in liquid YPGal media and mitochondria were isolated according to Meisinger and colleagues^58^. Equal amounts of mitochondrial proteins were loaded on SDS-PAGE, transferred to nitrocellulose membrane and detected by immunoblotting.

To investigate the effects of mIF3/Aim23 on mitochondrial translation, we followed incorporation of ^35^S-methionine in the presence of 0.2 mg/ml of the antibiotic cycloheximide that specifically inhibits cytoplasmic translation^32^ (Fig. 3A). *S. cerevisiae* mtDNA encodes only eight protein genes^4^, seven of which encode components of mitochondrial OXPHOS complexes, with the remaining gene encoding a ribosomal protein, Var1^4^. Therefore, we can follow synthesis of all the eight individual polypeptides by resolving them on an SDS PAGE gel.

The overall efficiency of mitochondrial protein synthesis in the *aim23*∆ strain is similar to that of wild type (Fig. 3A); a highly surprising observation given that the bacterial ortholog of mIF3/Aim23, IF3, is crucial for two steps of the ribosomal cycle: initiation and ribosome recycling. This result is in stark contrast to the near-complete inhibition of translation observed upon thermal inactivation of a temperature-sensitive version of *S. cerevisiae* mitochondrial mtRRF – a specialized factor mediating ribosomal recycling in bacteria^33^ and mitochondria^34^. The relative abundance of the mitochondrially synthesized proteins is, however, altered in the *aim23*∆ strain, affecting both the kinetics of protein production (Fig. 3A, Supplementary Figure 1) and the protein levels after a 5 minute-long (Fig. 3B) and 20 minute-long (Supplementary Figure 2) pulse labeling. To quantify the effects of knocking out *AIM23* we have normalized the relative expression levels, i.e. presented individual protein expression as a fraction of total expression. The kinetics of ^35^S-methionine incorporation has a pronounced biphasic nature (Fig. 3A, Supplementary Figure 1): during the initial linear phase up to around the 5 minute time point ^35^S-methionine incorporation into newly synthesized cytochrome *c* oxidase subunits Cox1, Cox2 is reduced about two times in the *aim23*∆ strain, while synthesis of ^35^S-methionine-labelled ATP synthase subunit Atp9 is promoted. In later time points (10, 15 and 20 minutes), labeling deviates from linearity and is saturating for some protein species (Supplementary Figure 1). As a result, in addition to the abovementioned effects on Cox1, Cox2 and Atp9, ^35^S-methionine labeling of nascent Atp6 and Atp8 is increased in the *aim23*∆ strain relative to the wild type, while labeling levels of Cox3 is decreased (compare Fig. 3B and Supplementary Figure 2; see also Supplementary Figure 1).

The observed imbalance in protein production can potentially be brought about by changes in transcription, translation, or stability of mRNA or protein. Several quality control systems in mitochondria recognize and degrade unfolded individual proteins or properly assembled proteins complexes^35^, convoluting the effects on synthesis and stability. We assessed protein stability by means of a chase experiment: after the initial labeling with ^35^S-methionine for 15 minutes, synthesis of radiolabeled proteins was stopped by the addition of an excess of ‘cold’ methionine and the levels of labeled proteins were followed over a 2 hour-long time course (Fig. 3C, see also Supplementary Figure 3 for two additional biological replicates). With the exception of Atp8 in the wild type strain, all other mitochondrial proteins are stable over the 120 minute-long time course, while ^35^S-methionine incorporation experiments show clear effects already after 5 minutes of labeling. This suggests that alterations in protein stability cannot be the cause for the unbalanced protein synthesis in the *aim23*∆ strain.

To assess the steady state levels of mitochondrially synthesized proteins, which are defined by both the rate of the protein’s synthesis and degradation, we performed Western blot detection of Cox1, Cox2, Cox3, Atp6, Atp9 and Cob along with mitochondrial outer membrane protein Porin 1 as a control (Fig. 3D, Supplementary Figure 4). The results are in agreement with the ^35^S-methionine incorporation data for cytochrome *c* oxidase subunits. Cox1 decrease being considerably more dramatic as judged by the Western blot. Lower Cox3 levels as detected by Western blot are in better agreement with the 20-minute ^35^S-methionine incorporation data (Supplementary Figures 1 and 2) than with 5 minute labeling time point (Fig. 3B). In the case of Atp6 and Atp9, the expression levels detected by Western blot are similar in wild type and the *aim23*∆ strains. A possible reason for the discrepancy between this result and the ^35^S-methionine labeling data could be the atypical biphasic ^35^S-methionine incorporation kinetics into newly synthesized Atp6 (Supplementary Figure 1). At up to 5 minutes the rate of ^35^S-Atp6 synthesis is similar in wild type and *aim23*∆ strains, and the differences observed in the later time points could reflect the differential depletion of cellular factors necessary for Atp6 synthesis in the two backgrounds rather then a true difference in protein production in the context of a live cell.

**Figure 4:**
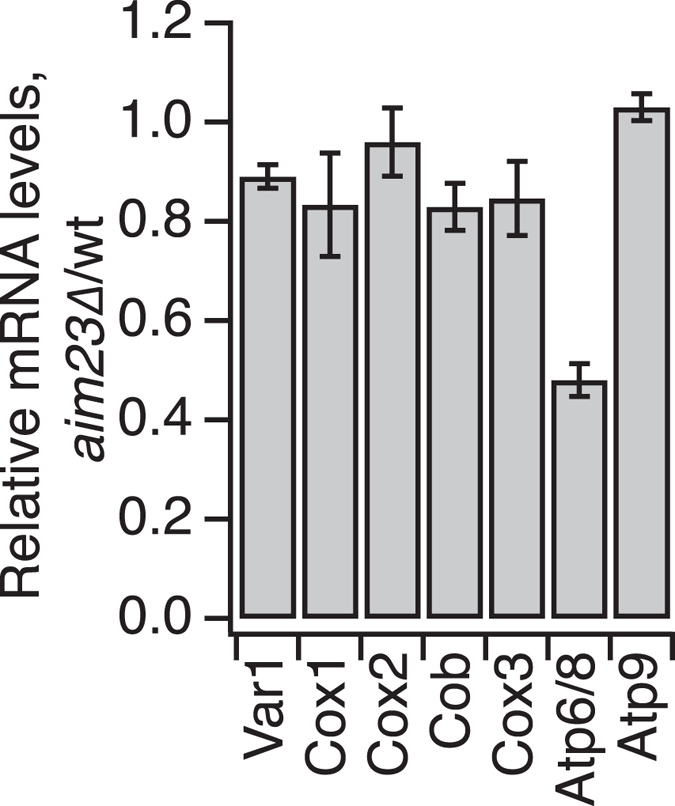
Northern blot analysis of all eight mitochondrially-encoded protein-coding mRNAs normalized to 21S rRNA. Atp6/8 designates a bicistronic mRNA encoding *ATP6* and *ATP8*.

**Figure 5:**
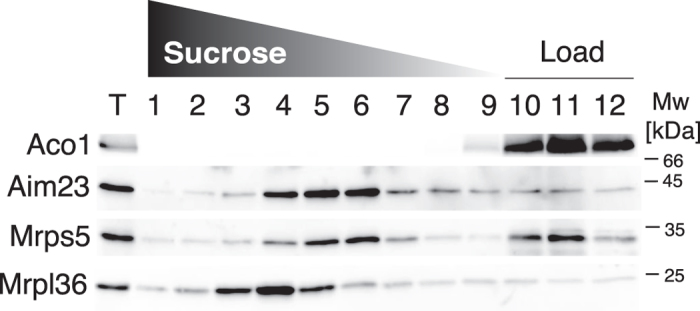
Aim23 specifically associates with the small subunit of mitochondrial ribosome. Mitochondria were lysed with 1% n-dodecyl β-D-maltoside in the presence of 100 mM KOAc leading to the dissociation of mitochondrial ribosomes into subunits, and the lysate was separated by centrifugation on a linear sucrose gradient as per Kehrein and colleagues^57^. Aconitase (Aco1) was used as soluble protein control and it stays in the top of the gradient. Separated ribosomal subunits migrate into the gradient and are detected using antibodies against small subunit protein Mrps5 and large subunit protein Mrpl36. T is a loading control corresponding to 10% of the starting material applied on the gradient, Mw is molecular weight in kDa.

Since the observed imbalance in mitochondrial protein production could, in principle, be caused by altered mRNA levels rather than by a direct effect on translation, we compared the mRNA levels in the wild type and *aim23*∆ strains using Northern blot hybridization (Fig. 4). With the exception of a lower level of the bicistronic mRNA encoding Atp6 and Atp8, we detect no significant differences in the mRNA levels between the wild type and *aim23*∆ strain. The decreased mRNA Atp6/Atp8 level is surprising, given that the expression of both proteins is increased in the *aim23*∆ strain (Fig. 3). Aim23 associates with the small subunit of the mitochondrial ribosome as demonstrated by Western blot analysis of lysed isolated *S. cerevisiae* mitochondria fractionated in a sucrose gradient under buffer conditions that induce separation of mitochondrial ribosomal subunits (Fig. 5). This further supports the direct involvement of Aim23 in mitochondrial protein synthesis.

## Discussion

Mitochondrial translation is of significant importance from a human health perspective; defects in mitochondrial translation are associated with a number of human genetic diseases^36^,^37^. It is also a potential drug target; the disabling of mitochondrial machinery with antibiotics may be a promising therapeutic tool in anti-cancer efforts^38^, but can also be a detrimental and undesirable side effect of antibacterial treatments^39^. *S. cerevisiae* is a valuable model organism for studying mitochondrial disease mechanisms and discovering therapies due to its amenability to mitochondrial and nuclear genome manipulation, as well as the ability to survive in the absence of functional mitochondrial oxidative phosphorylation (see recent review by Lasserre and colleagues^40^). Therefore, understanding the molecular mechanisms of mitochondrial protein synthesis and its regulation in this organism is of great importance for both basic and applied research.

Maintenance of the appropriate relative levels of mitochondrial expression is crucial for the assembly of functional OXPHOS complexes, and numerous auto-regulatory mechanisms are in place to ensure this balance in *S. cerevisiae* (reviewed by Fontanesi^41^). Assembly of the genome-encoded F_1_ subunit of F_1_F_0_ ATP synthase is the key regulator of the expression of Atp6 and Atp8 subunits^42^. Similarly, the expression of cytochrome *b*, Cob, a core component of complex III, is controlled by an analogous autoregulatory feedback mechanism^43^,^44^. Loss of mIF3/AIM23 does not, surprisingly, result in a general defect of translation; instead, it perturbs the stoichiometry of mitochondrially synthesized proteins (Fig. 3, Supplementary Figures 1 and 2). The mechanism behind the misbalance is unclear. It is principally possible that mIF3 acts as an mRNA-specific translational factor; however this is unlikely given that the core functions of the bacterial general translational factor IF3, i.e. subunit anti-association and stimulation of the initiator fMet-tRNA_i_^fMet^ binding to programmed ribosome, are preserved in human mIF3^19^. Therefore, we hypothesize that the misbalance is a consequence of the differential efficiency of ribosomal recruitment to different mRNAs in the absence of mIF3, resulting in a new ‘pecking order’ among the individual mRNAs competing for ribosomes.

The modest requirement for mIF3 in mitochondrial protein synthesis in *S. cerevisiae* is somewhat surprising given that IF3 is absolutely essential in *E. coli*^25^. At the same time, a disruption of the *mti3* gene encoding mtIF3 in *S. pombe* does not even lead to a severe phenotype^45^. *S. pombe* is a petite-negative yeast, i.e. it can not survive without functional mitochondria^46^, which, in turn absolutely requires functional mitochondrial translation^47^. Therefore, one can conclude that the absence of mIF3 does not abrogate mitochondrial translation in *S. pombe* either. Moreover, mIF3 is seemingly naturally missing in a handful of organisms, including yeast *Yarrowia lipolytica*^11^. All these lines of evidence suggest that mIF3 is, indeed, dispensable for mitochondrial translation, but the importance of the functionality of the protein varies from organism to organism.

The effects of mIF3 loss on the functionality of mammalian – and specifically human – mitochondria have not been studied. Mutations destabilizing human mIF3 mRNA are associated with Parkinson’s disease^48^,^49^,^50^. The connection between Parkinson’s and mtDNA instability is well established (for a review see^51^), which fits well with the elevated petite frequency in the *aim23*∆ *S. cerevisiae* strain, an indirect readout of mtDNA stability. Follow-up experiments in a mammalian system are necessary to directly address the role of mIF3 in humans.

## Methods

### Analysis of yeast growth rates on plates

D273-10B (*MATα mal*) and D273-10B *aim23*∆ (*MATα mal AIM23*::*kanMX4*) yeast strains were grown in liquid YPD medium (2% bacto-peptone, 1% yeast extract, 2% glucose) until OD_600_ of 3–4, washed with water and spotted onto solid YPGly (2% bacto-peptone, 1% yeast extract, 3% glycerol) or YPP (2% bacto-peptone, 1% yeast extract, 2% ethanol, 25 mM sodium phosphate buffer pH 6.2) media. 10× serial dilutions were used, starting from OD_600_ 0.1. Plates were incubated at 30 °C and scored at 24, 48 and 72 hours.

### Analysis of yeast growth rates in liquid cultures

Single colonies of D273-10B (*MATα mal*) and D273-10B *aim23*∆ (*MATα mal AIM23*::*kanMX4*) yeast strains were transferred from a YPD (2% bacto-peptone, 1% yeast extract, 2% glucose, 1.5% agar) agar plate to 5 mL of liquid medium of the same composition (minus agar) and were grown at 30 °C for 15–18 hours, reaching final OD_600_ of 4.0–5.0. Cells were gently pelleted, washed with water and inoculated into liquid YPGly media at OD_600_ of 0.1. Growth rates were monitored at 30 °C every 18 min for 4.5 days in a TECAN microplate reader equipped with a temperature control unit.

### Monitoring of mitochondrial translation *in vivo*

Pulse labelling of mitochondrial proteins with ^35^S-methionine was carried out in whole cells in the presence of 0.2 mg/ml cycloheximide according to Gouget and colleagues^32^. Equal amounts of total cell proteins were separated on a 17.5% PAGE gel, subjected to autoradiographic analysis and quantification using ImageJ^52^. A 20 minute-long incubation as the end-point in the ^35^S-methionine incorporation experiments was chosen as per Herrmann and colleagues^53^.

### Western blot analysis of isolated mitochondria

Equal amounts of mitochondrial proteins were loaded on SDS-PAGE, transferred to nitrocellulose membrane and immunodecorated with corresponding antisera (Cox1 1:300, Cox3 1:300, Tom70 1:3000, all gifted by Roland Lill; Cox2 1:3000, ABCAM; Atp6 1:10000, Atp9 1:5000, gifted by Marie-France Giraud; Porin 1 1:3000, ABCAM) and secondary anti-rabbit antibodies (ABCAM). Atp9 forms ring-like oligomers^54^ that are stable under standard conditions of preparation of the protein extracts for Western-blotting analysis. Therefore in order to promote the formation of Atp9 monomers total protein extracts were precipitated with 10% TCA, and then the pellets were resuspended in 10 mM Tris-HCl pH 7.0, 5% SDS (Marie-France Giraud, personal communication). Porin 1 was used as a control for equal loading.

### RNA detection by Northern blot hybridization

Mitochondria were isolated from 1 l of yeast cultures after cultivation in liquid YPGly media for 16 hours (starting OD_600_ of 0.5, final OD_600_ of 3 for wild type and 1.5 for *aim23*∆). Total RNA was extracted from mitochondria with TRIzol reagent (Life Technologies) according to the standard procedure. About 5 μg of RNA was separated on the 1% denaturating MOPS-formaldehyde agarose gel^55^ and transferred to the Hybond-N+ (GE Healthcare) membrane according to the manufacturer’s instructions. Membranes were hybridized with the oligonucleotide probes according to Mager-Heckel and colleagues^56^. Visualization was performed using Storm Imager 685 (GE Healthcare), and mRNA levels were normalized to 21S rRNA. Oligonucleotides used for Northern blotting, 5′-3′:

VAR1 GACCAATCCGGTGAACAACCGGATTGGC,

COX1 GCACCCATTGATAATACATAGTGAAAATGTCCCACCACGTAG,

COX2 AACTCAGAACATGCTCCATAGAAGACAC,

COX3 TACCAGCATAGAATACTGAACCATAAACAC,

COB AGTATTACCTCTTACTACACTTCTATCAGTA,

ATP6/8GAATCATTAATAAGAAACCATATGTTAATTGATTCATAAAATAAAATGGAACTAATTGTGGC,

ATP9 TACTAGGTCTTTAATTGATGGGTTTCTT,

21S rRNA CTATATTACCCTGTTATCCCTAGCGTAACT.

### Fluorescence microscopy of live yeast cells

Nuclear and mitochondrial DNA staining with DAPI (4′,6-diamidino-2-phenylindole) was performed according to the protocol of Amberg and colleagues^28^.

### Analysis of “petite” frequency

Yeast cultures were grown until OD_600_ of 3–4 in YPD liquid medium, diluted with fresh medium and grown to OD_600_ of 0.8–1, washed with water and plated onto solid YPGly media supplemented with 0.1% glucose. The number of small colonies (“petites”) was scored after 5 days of growth at 30 °C and expressed as a percentage of the total colony count.

### Respiration rate measurements using a Clark electrode

Oxygen consumption by intact yeast cells was monitored in phosphate-buffered saline buffer using a Clark electrode (Hansatech Instruments) at 30 °C. Cells were grown in YPD media until late-log phase, gently pelleted and thoroughly washed with water, inoculated to YPGly media and grown to OD_600_ of 1, collected by gentle centrifugation, washed several times with PBS and subjected to direct oxymetry.

### Detection of Aim23 associated with isolated *S. cerevisiae* mitochondrial ribosomes

Mitochondria were lysed by addition of 1% n-dodecyl β-D-maltoside in the presence of 100 mM KOAc to separate the subunits and subjected to centrifugation on a linear sucrose gradient (1–0.3 M) according to Kehrein *et al.*^57^. Anti-Aim23 antibodies were raised against recombinantly expressed and purified Aim23.

## Additional Information

**How to cite this article**: Kuzmenko, A. *et al.* Aim-less translation: loss of *Saccharomyces cerevisiae* mitochondrial translation initiation factor mIF3/Aim23 leads to unbalanced protein synthesis. *Sci. Rep.* **6**, 18749; doi: 10.1038/srep18749 (2016).

## Supplementary Material

Supplementary Information

## References

[b1] McBride H. M., Neuspiel M. & Wasiak S. Mitochondria: more than just a powerhouse. Curr Biol 16, R551–60 (2006). 16860735 10.1016/j.cub.2006.06.054

[b2] Gray M. W. Mitochondrial evolution. Cold Spring Harb Perspect Biol 4, a011403 (2012). 22952398 10.1101/cshperspect.a011403 PMC3428767

[b3] Barbrook A. C., Howe C. J., Kurniawan D. P. & Tarr S. J. Organization and expression of organellar genomes. Philos Trans R Soc Lond B Biol Sci 365, 785–97 (2010). 20124345 10.1098/rstb.2009.0250 PMC2817230

[b4] Foury F., Roganti T., Lecrenier N. & Purnelle B. The complete sequence of the mitochondrial genome of Saccharomyces cerevisiae. FEBS Lett 440, 325–31 (1998). 9872396 10.1016/s0014-5793(98)01467-7

[b5] Anderson S. *et al.* Sequence and organization of the human mitochondrial genome. Nature 290, 457–65 (1981). 7219534 10.1038/290457a0

[b6] Kehrein K., Bonnefoy N. & Ott M. Mitochondrial protein synthesis: efficiency and accuracy. Antioxid Redox Signal 19, 1928–39 (2013). 23088322 10.1089/ars.2012.4896

[b7] Brown A. *et al.* Structure of the large ribosomal subunit from human mitochondria. Science 346, 718–22 (2014). 25278503 10.1126/science.1258026 PMC4246062

[b8] Amunts A., Brown A., Toots J., Scheres S. H. & Ramakrishnan V. Ribosome. The structure of the human mitochondrial ribosome. Science 348, 95–8 (2015). 25838379 10.1126/science.aaa1193 PMC4501431

[b9] Greber B. J. *et al.* Ribosome. The complete structure of the 55S mammalian mitochondrial ribosome. Science 348, 303–8 (2015). 25837512 10.1126/science.aaa3872

[b10] Greber B. J. *et al.* The complete structure of the large subunit of the mammalian mitochondrial ribosome. Nature 515, 283–6 (2014). 25271403 10.1038/nature13895

[b11] Atkinson G. C. *et al.* Evolutionary and genetic analyses of mitochondrial translation initiation factors identify the missing mitochondrial IF3 in S. cerevisiae. Nucleic Acids Res 40, 6122–34 (2012). 22457064 10.1093/nar/gks272 PMC3401457

[b12] Fox T. D. Mitochondrial protein synthesis, import, and assembly. Genetics 192, 1203–34 (2012). 23212899 10.1534/genetics.112.141267 PMC3512135

[b13] Ohtsuki T., Sato A., Watanabe Y. & Watanabe K. A unique serine-specific elongation factor Tu found in nematode mitochondria. Nat Struct Biol 9, 669–73 (2002). 12145639 10.1038/nsb826

[b14] Atkinson G. C. The evolutionary and functional diversity of classical and lesser-known cytoplasmic and organellar translational GTPases across the tree of life. BMC Genomics 16, 78 (2015). 25756599 10.1186/s12864-015-1289-7 PMC4342817

[b15] Tsuboi M. *et al.* EF-G2mt is an exclusive recycling factor in mammalian mitochondrial protein synthesis. Mol Cell 35, 502–10 (2009). 19716793 10.1016/j.molcel.2009.06.028

[b16] Akabane S., Ueda T., Nierhaus K. H. & Takeuchi N. Ribosome rescue and translation termination at non-standard stop codons by ICT1 in mammalian mitochondria. PLoS Genet 10, e1004616 (2014). 25233460 10.1371/journal.pgen.1004616 PMC4169044

[b17] Kuzmenko A. *et al.* Mitochondrial translation initiation machinery: conservation and diversification. Biochimie 100, 132–40 (2014). 23954798 10.1016/j.biochi.2013.07.024 PMC3978653

[b18] Herrmann J. M., Woellhaf M. W. & Bonnefoy N. Control of protein synthesis in yeast mitochondria: the concept of translational activators. Biochim Biophys Acta 1833, 286–94 (2013). 22450032 10.1016/j.bbamcr.2012.03.007

[b19] Koc E. C. & Spremulli L. L. Identification of mammalian mitochondrial translational initiation factor 3 and examination of its role in initiation complex formation with natural mRNAs. J Biol Chem 277, 35541–9 (2002). 12095986 10.1074/jbc.M202498200

[b20] Liao H. X. & Spremulli L. L. Initiation of protein synthesis in animal mitochondria. Purification and characterization of translational initiation factor 2. J Biol Chem 266, 20714–9 (1991). 1939122

[b21] Hess D. C. *et al.* Computationally driven, quantitative experiments discover genes required for mitochondrial biogenesis. PLoS Genet 5, e1000407 (2009). 19300474 10.1371/journal.pgen.1000407 PMC2648979

[b22] Elvekrog M. M. & Gonzalez R. L. Jr. Conformational selection of translation initiation factor 3 signals proper substrate selection. Nat Struct Mol Biol 20, 628–33 (2013). 23584454 10.1038/nsmb.2554 PMC3648635

[b23] Zavialov A. V., Hauryliuk V. V. & Ehrenberg M. Splitting of the posttermination ribosome into subunits by the concerted action of RRF and EF-G. Mol Cell 18, 675–86 (2005). 15949442 10.1016/j.molcel.2005.05.016

[b24] Peske F., Rodnina M. V. & Wintermeyer W. Sequence of steps in ribosome recycling as defined by kinetic analysis. Mol Cell 18, 403–12 (2005). 15893724 10.1016/j.molcel.2005.04.009

[b25] Olsson C. L., Graffe M., Springer M. & Hershey J. W. Physiological effects of translation initiation factor IF3 and ribosomal protein L20 limitation in Escherichia coli. Mol Gen Genet 250, 705–14 (1996). 8628231 10.1007/BF02172982

[b26] Dimitrov L. N., Brem R. B., Kruglyak L. & Gottschling D. E. Polymorphisms in multiple genes contribute to the spontaneous mitochondrial genome instability of Saccharomyces cerevisiae S288C strains. Genetics 183, 365–83 (2009). 19581448 10.1534/genetics.109.104497 PMC2746160

[b27] Polakis E. S. & Bartley W. Changes in the enzyme activities of Saccharomyces cerevisiae during aerobic growth on different carbon sources. Biochem J 97, 284–97 (1965). 16749116 10.1042/bj0970284 PMC1264573

[b28] Amberg D. C., Burke D. J. & Strathern J. N. Yeast Vital Stains: DAPI Stain of Nuclear and Mitochondrial DNA. CSH Protoc 2006 (2006). 10.1101/pdb.prot4163 22485564

[b29] Chen X. J. & Clark-Walker G. D. The petite mutation in yeasts: 50 years on. Int Rev Cytol 194, 197–238 (2000). 10494627 10.1016/s0074-7696(08)62397-9

[b30] Dujon B. in The Molecular Biology of the Yeast Saccharomyces: Life Cycle and Inheritance (eds. Strathern J. N., Jones E. W. & Broach J. R.) 505–635 (Cold Spring Harbor Laboratory Press, Cold Spring Harbor, NY, 1981).

[b31] Silva A. M. & Oliveira P. J. Evaluation of respiration with clark type electrode in isolated mitochondria and permeabilized animal cells. Methods Mol Biol 810, 7–24 (2012). 22057558 10.1007/978-1-61779-382-0_2

[b32] Gouget K., Verde F. & Barrientos A. *In vivo* labeling and analysis of mitochondrial translation products in budding and in fission yeasts. Methods Mol Biol 457, 113–24 (2008). 19066022 10.1007/978-1-59745-261-8_8

[b33] Janosi L., Hara H., Zhang S. & Kaji A. Ribosome recycling by ribosome recycling factor (RRF)–an important but overlooked step of protein biosynthesis. Adv Biophys 32, 121–201 (1996). 8781287 10.1016/0065-227x(96)84743-5

[b34] Teyssier E. *et al.* Temperature-sensitive mutation in yeast mitochondrial ribosome recycling factor (RRF). Nucleic Acids Res 31, 4218–26 (2003). 12853640 10.1093/nar/gkg449 PMC165964

[b35] Sekine S. & Ichijo H. Mitochondrial proteolysis: its emerging roles in stress responses. Biochim Biophys Acta 1850, 274–80 (2015). 25459516 10.1016/j.bbagen.2014.10.012

[b36] Boczonadi V. & Horvath R. Mitochondria: impaired mitochondrial translation in human disease. Int J Biochem Cell Biol 48, 77–84 (2014). 24412566 10.1016/j.biocel.2013.12.011 PMC3988845

[b37] Pearce S., Nezich C. L. & Spinazzola A. Mitochondrial diseases: translation matters. Mol Cell Neurosci 55, 1–12 (2013). 22986124 10.1016/j.mcn.2012.08.013

[b38] Skrtic M. *et al.* Inhibition of mitochondrial translation as a therapeutic strategy for human acute myeloid leukemia. Cancer Cell 20, 674–88 (2011). 22094260 10.1016/j.ccr.2011.10.015 PMC3221282

[b39] Singh R., Sripada L. & Singh R. Side effects of antibiotics during bacterial infection: mitochondria, the main target in host cell. Mitochondrion 16, 50–4 (2014). 24246912 10.1016/j.mito.2013.10.005

[b40] Lasserre J. P. *et al.* Yeast as a system for modeling mitochondrial disease mechanisms and discovering therapies. Dis Model Mech 8, 509–526 (2015). 26035862 10.1242/dmm.020438 PMC4457039

[b41] Fontanesi F. Mechanisms of mitochondrial translational regulation. IUBMB Life 65, 397–408 (2013). 23554047 10.1002/iub.1156

[b42] Rak M. & Tzagoloff A. F1-dependent translation of mitochondrially encoded Atp6p and Atp8p subunits of yeast ATP synthase. Proc Natl Acad Sci USA 106, 18509–14 (2009). 19841266 10.1073/pnas.0910351106 PMC2774022

[b43] Hildenbeutel M. *et al.* Assembly factors monitor sequential hemylation of cytochrome b to regulate mitochondrial translation. J Cell Biol 205, 511–24 (2014). 24841564 10.1083/jcb.201401009 PMC4033779

[b44] Gruschke S. *et al.* The Cbp3-Cbp6 complex coordinates cytochrome b synthesis with bc (1) complex assembly in yeast mitochondria. J Cell Biol 199, 137–50 (2012). 23007649 10.1083/jcb.201206040 PMC3461508

[b45] Kim D. U. *et al.* Analysis of a genome-wide set of gene deletions in the fission yeast Schizosaccharomyces pombe. Nat Biotechnol 28, 617–23 (2010). 20473289 10.1038/nbt.1628 PMC3962850

[b46] Schafer B. Genetic conservation versus variability in mitochondria: the architecture of the mitochondrial genome in the petite-negative yeast Schizosaccharomyces pombe. Curr Genet 43, 311–26 (2003). 12739049 10.1007/s00294-003-0404-5

[b47] Chiron S., Suleau A. & Bonnefoy N. Mitochondrial translation: elongation factor tu is essential in fission yeast and depends on an exchange factor conserved in humans but not in budding yeast. Genetics 169, 1891–901 (2005). 15695360 10.1534/genetics.104.037473 PMC1449603

[b48] Behrouz B. *et al.* Mitochondrial translation initiation factor 3 polymorphism and Parkinson’s disease. Neurosci Lett 486, 228–30 (2010). 20887776 10.1016/j.neulet.2010.09.059

[b49] Anvret A. *et al.* Possible involvement of a mitochondrial translation initiation factor 3 variant causing decreased mRNA levels in Parkinson’s disease. Parkinsons Dis 2010, 491751 (2010). 20976088 10.4061/2010/491751 PMC2957232

[b50] Abahuni N. *et al.* Mitochondrial translation initiation factor 3 gene polymorphism associated with Parkinson’s disease. Neurosci Lett 414, 126–9 (2007). 17267121 10.1016/j.neulet.2006.12.053

[b51] Pickrell A. M. & Youle R. J. The roles of PINK1, parkin, and mitochondrial fidelity in Parkinson’s disease. Neuron 85, 257–73 (2015). 25611507 10.1016/j.neuron.2014.12.007 PMC4764997

[b52] Schneider C. A., Rasband W. S. & Eliceiri K. W. NIH Image to ImageJ: 25 years of image analysis. Nat Methods 9, 671–5 (2012). 22930834 10.1038/nmeth.2089 PMC5554542

[b53] Herrnmann J., Fölsch H., Neupert W. & Stuart R. in Cell biology. A laboratory handbook (ed. Celis J.) 538–544 (Academic Press, 1994).

[b54] Stock D., Leslie A. G. & Walker J. E. Molecular architecture of the rotary motor in ATP synthase. Science 286, 1700–5 (1999). 10576729 10.1126/science.286.5445.1700

[b55] Brown T., Mackey K. & Du T. Analysis of RNA by northern and slot blot hybridization. Curr Protoc Mol Biol Chapter 4, Unit 4 9 (2004). 10.1002/0471142727.mb0409s67 18265351

[b56] Mager-Heckel A. M. *et al.* The analysis of tRNA import into mammalian mitochondria. Methods Mol Biol 372, 235–53 (2007). 18314730 10.1007/978-1-59745-365-3_17

[b57] Kehrein K. *et al.* Organization of Mitochondrial Gene Expression in Two Distinct Ribosome-Containing Assemblies. Cell Rep (2015). 10.1016/j.celrep.2015.01.012 25683707

[b58] Meisinger C., Pfanner N. & Truscott K. N. Isolation of yeast mitochondria. Methods Mol Biol 313, 33–9 (2006). 16118422 10.1385/1-59259-958-3:033

