# Supplementary material for: Aim-less translation: loss of Saccharomyces cerevisiae mitochondrial translation initiation factor mIF3/Aim23 leads to unbalanced protein synthesis
Source: Sci Rep. 2016 Jan 5;6:18749. doi: 10.1038/srep18749 (PMC4700529; doi:10.1038/srep18749)
Supplement: Supplementary Information [file srep18749-s1.pdf]

Supplementary Information

Aim-less translation: loss of *Saccharomyces cerevisiae* mitochondrial translation initiation factor mIF3/Aim23 leads to unbalanced protein synthesis

Anton Kuzmenko[1,2], Ksenia Derbikova[2], Roger Salvatori[3], Stoyan Tankov[1], Gemma C. Atkinson[1,4,5], Tanel Tenson[1], Martin Ott[3], Piotr Kamenski[2,*] and Vasili Hauryliuk[1,4,5,*]

[1]University of Tartu, Institute of Technology, Nooruse 1, 50411 Tartu, Estonia
[2]Molecular Biology Department, Faculty of Biology, M.V. Lomonosov Moscow State University, 1/12 Leninskie Gory, 119991 Moscow, Russia
[3]Department of Biochemistry and Biophysics, Center for Biomembrane Research, Stockholm University, SE-106 91 Stockholm, Sweden
[4]Department of Molecular Biology, Umeå University, Building 6K, 6L University Hospital Area, SE-901 87 Umeå, Sweden
[5]Laboratory for Molecular Infection Medicine Sweden (MIMS), Umeå University, Building 6K and 6L, University Hospital Area, SE-901 87 Umeå, Sweden

[*] denotes the corresponding authors

Contact details of corresponding authors:

Piotr Kamenski: piotr.kamenski@gmail.com, +74959395485
Vasili Hauryliuk: vasili.hauryliuk@molbiol.umu.se, +46907850807

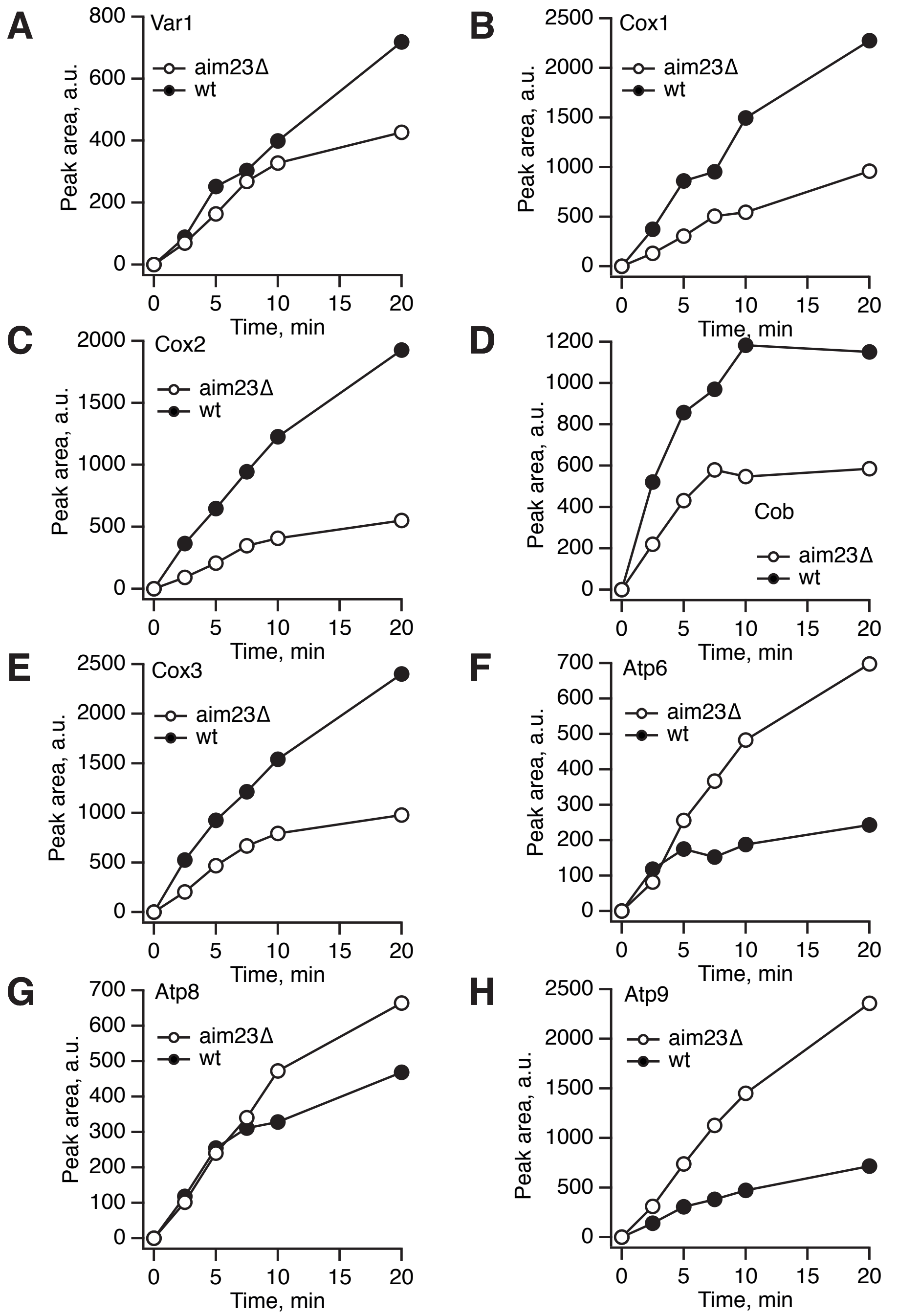

Supplementary Figure 1 | Kinetics of ^{35}S-methionine incorporation in newly synthesized mitochondrial proteins. The experiments were performed using a congenic set of wild type and *aim23Δ* cells in a BY4741 background. The raw data are presented in **Figure 3A**. ^{35}S-methionine pulse labeling was carried out as per Gouget and colleagues[1]

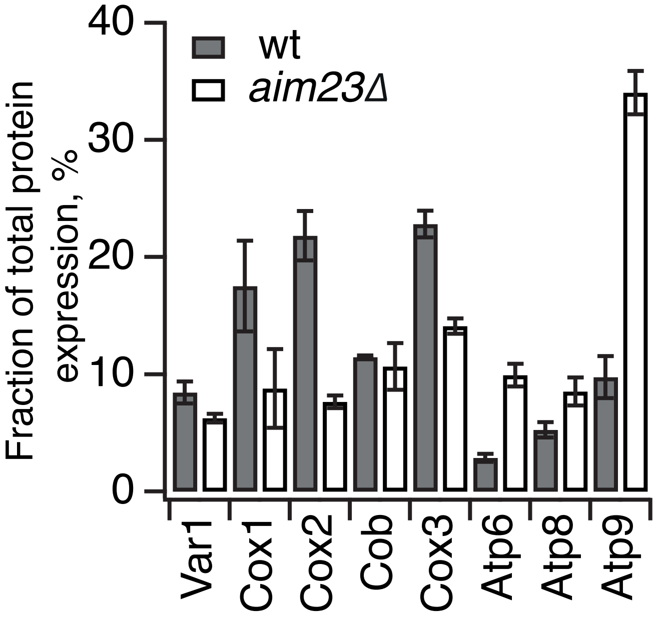

Supplementary Figure 2 | Levels of mitochondrially-encoded proteins after 20 min labeling with ^{35}S-methionine. The relative expression is normalized to total expression of mitochondrially encoded protein genes.

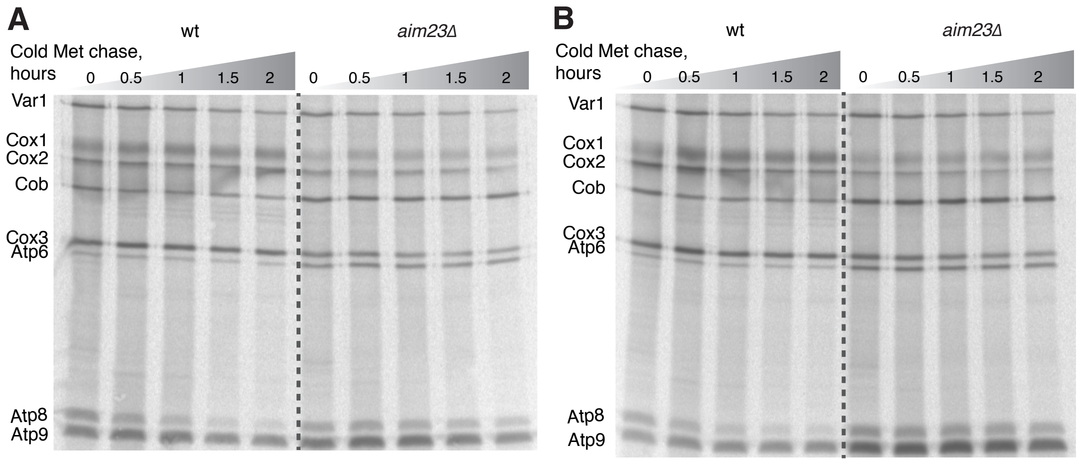

Supplementary Figure 3 | Turnover of mitochondrially synthesised proteins in wild type and *aim23Δ* strains. After 15 minutes of ^{35}S methionine pulse labeling was carried out as per Gouget and colleagues[1], the labeling reaction was stopped by the addition of cold methionine (final concentration of 80 mM) and puromycin (final concentration of 4 μg/ml). Samples were collected after the indicated time points, proteins were resolved on SDS PAGE and visualized by radioautography. Panels (**A**) and (**B**) represent two biological replicates.

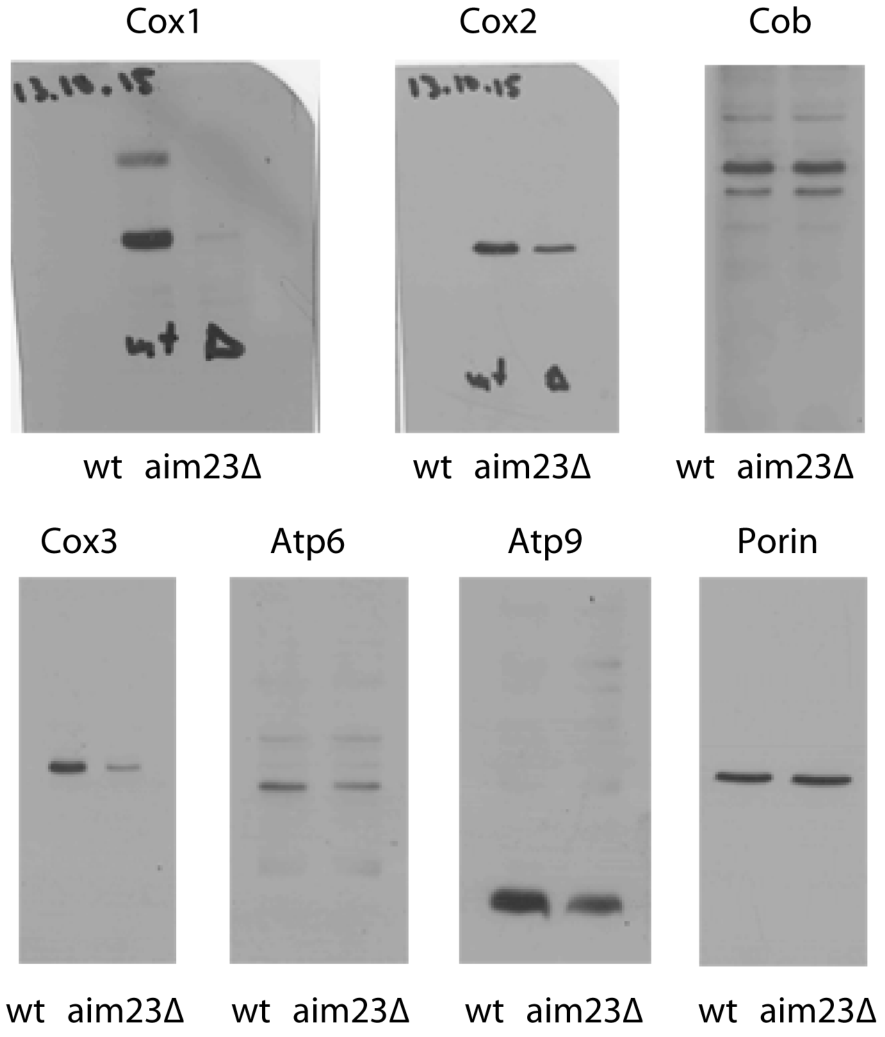

Supplementary Figure 4 | Unprocessed pictures of Western blots used in this work (see Figure 3D).

Supplementary References:

1. Gouget, K., Verde, F. & Barrientos, A. In vivo labeling and analysis of mitochondrial translation products in budding and in fission yeasts. *Methods Mol Biol* **457**, 113-24 (2008).
